# The aqueous extract of *Artemisia Absinthium* L. stimulates HO-1/MT-1/Cyp450 signaling pathway via oxidative stress regulation induced by aluminium oxide nanoparticles (α and γ) animal model

**DOI:** 10.1186/s12906-023-04121-6

**Published:** 2023-09-05

**Authors:** Esmaeil Karami, Zahra Goodarzi, Seyed Jamaleddin Shahtaheri, Mehrafarin Kiani, Mohammad Faridan, Mahmoud Ghazi-Khansari

**Affiliations:** 1 Department of Occupational Health Engineering, School of Health, Tehran University of Medical Sciences, Tehran, Iran; 2 Department of Occupational Health Engineering, Faculty of Medical Sciences, Tarbiat Modares University, Tehran, Iran; 3 Center for Water Quality Research, Institute for Environmental Research, Tehran University of Medical Sciences, Tehran, Iran; 4 Department of Anatomical Sciences, Faculty of Medical Sciences, Tarbiat Modares University, Tehran, Iran; 5 Department of Occupational Health and Safety at Work Engineering, Environmental Health Research CenterLorestan University of Medical Sciences, Khorramabad, Iran; 6 Department of Pharmacology, School of Medicine, Tehran University of Medical Sciences, Tehran, Iran

**Keywords:** Oxidative stress, Al_2_O_3_ NPs, *Artemisia Absinthium* L, Histopathology, Gene expression

## Abstract

**Background:**

This research aimed to evaluate the protective effects of *Artemisia Absinthium L*. (Abs) against liver damage induced by aluminium oxide nanoparticles (Al_2_O_3_ NPs) in rats, including both structural and functional changes associated with hepatotoxicity.

**Methods:**

Thirty-six rats were randomly divided into six groups (*n* = 6). The first group received no treatment. The second group was orally administered Abs at a dose of 200 mg/kg/b.w. The third and fifth groups were injected intraperitoneally with γ-Al_2_O_3_ NPs and α-Al_2_O_3_ NPs, respectively, at a dose of 30 mg/kg/b.w. The fourth and sixth groups were pre-treated with oral Abs at a dose of 200 mg/kg/b.w. along with intraperitoneal injection of γ-Al_2_O_3_ NPs and α-Al_2_O_3_ NPs, respectively, at a dose of 30 mg/kg/b.w.

**Results:**

Treatment with γ-Al_2_O_3_ NPs resulted in a significant decrease (*P* < 0.05) in total body weight gain, relative liver weight to body weight, and liver weight in rats. However, co-administration of γ-Al_2_O_3_ NPs with Abs significantly increased body weight gain (*P* < 0.05). Rats treated with Al_2_O_3_ NPs (γ and α) exhibited elevated levels of malondialdehyde (MDA), inducible nitric oxide synthase (iNOS), alanine transaminase (ALT), and aspartate aminotransferase (AST). Conversely, treatment significantly reduced glutathione peroxidase (GPx), catalase (CAT), total superoxide dismutase (T-SOD), and total antioxidant capacity (TAC) levels compared to the control group. Furthermore, the expression of heme oxygenase-1 (HO-1) and metallothionein-1 (MT-1) mRNAs, cytochrome P450 (CYP P450) protein, and histopathological changes were significantly up-regulated in rats injected with Al_2_O_3_ NPs. Pre-treatment with Abs significantly reduced MDA, AST, HO-1, and CYP P450 levels in the liver, while increasing GPx and T-SOD levels compared to rats treated with Al_2_O_3_ NPs.

**Conclusion:**

The results indicate that Abs has potential protective effects against oxidative stress, up-regulation of oxidative-related genes and proteins, and histopathological alterations induced by Al_2_O_3_ NPs. Notably, γ-Al_2_O_3_ NPs exhibited greater hepatotoxicity than α-Al_2_O_3_ NPs.

## Introduction

Al_2_O_3_ nanoparticles (NPs) exist in various polymorphs, including γ-Al_2_O_3_ NPs and α-Al_2_O_3_ NPs phases, and have numerous applications across different industries [1]. In recent years, Al_2_O_3_ NPs have become widely used worldwide, particularly in medical, domestic, and industrial sectors such as catalysis, structural ceramics, polymer modification, and textile functionalization [2, 3]. The γ-Al_2_O_3_ NPs are commonly used as coatings, catalysts, soft abrasives, and adsorbents due to their smaller size, larger surface area, and lower melting point. On the other hand, the α-phase of these NPs possesses higher specific strength and is utilized in thermal insulating materials for its superior insulating properties [1, 4, 5].

However, the potential risks associated with the increasing utilization of Al_2_O_3_ NPs have not been thoroughly investigated yet. Recently, experiments have shown that exposure to Al_2_O_3_ NPs may have negative effects on humans [6, 7] and animals [8]. In vivo, research has demonstrated that exposure to Al_2_O_3_ NPs can induce hepatotoxicity in rats and lead to an inflammatory response, oxidative stress, and alterations in the antioxidant defense system [9–11]. The liver appears to be a target organ for identifying the adverse effects of in vivo exposure to Al_2_O_3_ NPs [12]. Prabhakar et al. reported that acute oral of Al_2_O_3_ NPs could induce changes in antioxidant status and generate oxidative stress [11].

Previous studies have also shown various harmful effects of Al_2_O_3_ NPs, including membrane and genotoxic damage [13], apoptosis [14], phagocytic dysfunction in alveolar macrophages [15, 16], cytotoxic [17], mitochondrial dysfunction [18], as well as DNA and protein damage, in both human and animal models.

Researchers have attributed the toxicity of Al_2_O_3_ NPs to their potential role in excessive production of reactive oxygen species (ROS) [19]. Further research also suggests that the oxidative stress caused by ROS regulates the generation of MT-1 and HO-1 [20, 21]. Oxidative stress rapidly induces the production of the MT-1 gene, which protects mammalian cells against oxidative damage in HEPA cells and reduces the number of intracellular oxygen radicals. Hepatocytes express a set of highly specific CYP450 biotransforming enzymes that convert various xenobiotics and endogenous substances into inactive or toxic compounds [22].

Currently, herbal medications and nutritional supplements are widely used worldwide to treat liver conditions [23]. Plants have long been recognized as significant sources of exogenous antioxidants and viable means to prevent oxidative damage caused by ROS [24, 25].

*Artemisia Absinthium L.* (Abs), commonly known as wormwood, is a perennial herbaceous and aromatic plant [26] with notable properties such as antimalarial [24], antibacterial [27], antiulcer [28], antitumor [29], antidepressant [30], antioxidant [31], neuroprotective [32], and hepatoprotective [33] effects.

Abs has been reported to possess free radical scavenging activity in both in vitro and in vivo experiments [34, 35].

Phytochemical studies have revealed that Abs contains essential oil, bitter sesquiterpenoid lactones, flavonoids, azulenes, phenolic acids, tannins and lignans [36]. Several flavonol-3-glycosides including quercetin, isorhamnetin, patuletin, and spinacetin derivatives, have been extracted from Abs leaves. In vitro studies have shown that Abs extracts exhibit strong antiradical and antioxidant activity, with high contents of total phenolic compounds and total flavonoids [35].

Abs aqueous extracts have demonstrated hepatoprotective effects against carbon tetrachloride toxicity, suggesting its association with antioxidant function [23]. Additionally, previous research has reported the hepatoprotective and nephroprotective activity of Abs against diclofenac-induced toxicity in rats [37]. Khoroubi et al. demonstrated that Abs extract not only restore the activity of the enzymes disrupted by lead exposure but also effectively prevented lipid peroxidation [38].

Due to its caffeoylquinic acid content, Abs has been considered a potential and promising antioxidant agent for mitochondria-targeted medicine in the treatment of oxidative stress-related degenerative diseases and cancers. In vitro experiments have yielded promising results, indicating that caffeoylquinic-acid-rich fractions derived from cultivated Artemisia species herb extracts can modulate mitochondrial function, exhibit antioxidant activity, and reduce cytochrome c levels through various mechanisms of action [39].

Although most prior studies have focused on the in vitro effects of Abs, further research and in vivo studies using animal models are necessary to better understand its mechanism of action. Therefore, this study aims to evaluate the possible effects of an aqueous extract of Abs on the antioxidant status and its protective ability against ROS, reactive nitrogen species (RNS), and gene expression in rats exposed to Al_2_O_3_ NPs.

## Materials and methods

### Plant collection and extraction method

First, the leaves and aerial flowers of the Abs plant were collected from the mountains of northern Tehran, Iran, in September 2021. All parts of the plant (5 cm from the end of the stem, approximately 50% leaves and stems, and 50% flowers) were dried at 25 °C in the shade before being pulverized through mechanical milling.

Since the poisonous chemicals in Abs (most notably thujone) are generally lipophilic in nature, water is the best solvent for reducing the toxicity of the extracts to the safe levels [40]. Therefore, in this study, we used an aqueous extract of the Abs plant.

2 litters of distilled water were added to 200 g of the obtained powder in an Erlenmeyer flask at room temperature. The flask's cap was secured, and the mixture was held there for 12 h. The flask's content was then transferred in a rotary apparatus at 60°C with an average rotation speed of 60 min. Finally, the mixture was filtered using a Whatman filter paper before being lyophilized in a freezer dryer. The remaining plant material was collected (yield 31 g) and stored at -20°C [23, 38].

### Chemicals

The α-Al_2_O_3_ NPs (80 nm, 100% alpha, white, hydrophilic, purity 99%, SSA: > 15 m^2^/g, density 3.97 g/cm^3^ with rhombohedral crystallographic structure) and γ-Al_2_O_3_ NPs (20 nm, white, 99% purity, SSA: > 138 m^2^/g, density 3890 kg/m^3^, almost spherical morphology) were purchased from US Research Nanomaterials Inc., Houston, TX USA (CAS No 1344–28-1, USA). Additionally, all the ultra-pure chemicals used in this research were purchased from Merck Company. MDA (CAS No. ZB-MDA-A96A), GPX (CAS No. ZB-GPX-A96), T-SOD (CAS No. 706002), TAC (CAS No. ZB-TAC-A96) CAT (CAS No. 707002), iNOS (CAS No. ZB-10740C-R9648) were assessed using ELISA kits (Zellbio Germany) and cDNA kit (Thermo scientific, US, K1622). For immunohistochemical assays, the following reagents were used: TBS 1X solution (Sigma-T5912), PBS (Sigma-P4417), DAPI (Sigma- D9542) Normal Goat Serum (10%) (G9023-Sigma), Triton 3% (Sigma-T8787), Alexa Fluor 488 (Elabscience, China), CYP450: GTX15616, Secondary antibodies (mouse): orb688924. SYBR Green master mix (Addbio Co., Korea) was utilized, following specific standards for each marker. AST and ALT were assessed using related kits (Pars Azmun, Tehran, Iran).

### Particle characterization

The morphology, particle size, hydrodynamic diameter of nanoparticles in solution, and crystal structure of both α-Al_2_O_3_ 80 nm and γ-Al_2_O_3_ 20 nm NPs were characterized using transmission electron microscopy (TEM), X-ray diffraction (XRD) patterns, and dynamic light scattering (DLS).

### DPPH radical-scavenging activity

To determine the free radical scavenging activity of the extract, the stable radical 1,1-diphenyl-2-picryl hydrazyl (DPPH) was considered. The assay conditions were as follows: 2.5 mg of extract and 1 ml of DPPH solution (300 µM); a total of 5 different concentrations with equal volumes, were prepared. After a residence time of 15 min at room temperature in darkness, the adsorption value was recorded at 517 nm for intervals ranging from 10 s to 2 min of reaction time in a UV–Vis spectrophotometer. The experiment was repeated three times. Butylated hydroxyanisole (BHA) and quercetin were used as standard controls. The IC50 values represent the concentration of the sample required to scavenge 50% of DPPH free radicals [30].

### Experimental animals

In accordance with the protocols for conducting scientific experiments on laboratory animals, a total of 36 adult Wistar rats weighing between 180 and 220 g were housed in groups of six per cage. The rats were kept in a controlled environment with a temperature of 22 ± 2°C, humidity of 55 ± 5%, and a 12-h light/dark cycle. They had free access to standard food and water throughout the experiment. Prior to commencing the study, the protocols were approved by the Animal Ethics Committee of Tehran University of Medical Sciences, Tehran, Iran, under permit number IR.TUMS.SPH.REC.1399.214.

### Study design

The rats were randomly assigned to six groups as follows: The first group served as normal control and was orally given deionized water for 15 days, and intraperitoneally injected with deionized water for 14 days. The Second group was pre-treated with Abs (200 mg/kg, oral gavage) for 15 days. Third and Fifth groups, respectively injected 30mg/kg of γ and α-Al_2_O_3_ NPs intraperitoneally for 14 days. Fourth and sixth groups were treated with Abs plus γ-Al_2_O_3_ NPs and Abs plus α-Al_2_O_3_ NPs respectively. Abs was administered by oral gavage for 15 days, starting one day before intraperitoneal administration of γ and α-Al_2_O_3_ NPs [On the second day, rats were administered Abs 60 min before receiving Al_2_O_3_ NPs].

The doses for Al_2_O_3_ NPs (γ and α) were determined based on the data from a pilot experiment and similar previous studies [41]. Both of these compounds were dissolved in deionised water and administered to the rats on a daily basis for 14 days. The rats that were treated with Al_2_O_3_ NPs (γ and α) received oral gavage of distilled water as well. The dose of Abs (200 mg/kg/b.w) was determined based on optimum Abs dosage from previous research with maximal efficacy [23, 38]. The rats were observed on a daily basis, and no animal died during the course of performing the experiment. Moreover, all rats received the treatments in the following timeline as well.
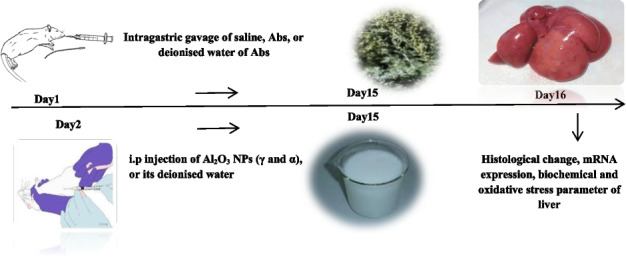


### Measuring the weight of body and liver

The values of initial and final body weight, body weight gain (Eq. 1), and relative body weight gain (Eq. 2) were regularly recorded. At the end of the experiment, the values of the final liver weight of each rat after being sacrificed and the total liver weight/final body weight ratio (g/100g) (Eq. 3) were also determined.

1 $$Body\ weight\ gain\ =\ Final\ body\ weight-Initial\ body\ weight$$

2 $$Relative\ body\ weight\ gain\ =\frac{Body\ weight\ gain}{Final\ body\ weight}$$

3 $$Relative\ weight\ of\ liver\ to\ body\ weight \left(\frac{g}{100g}\right)=\frac{Total\ liver\ weight}{Final\ body\ weight}\times 100$$

### Collecting the blood samples and liver tissues

At the end of the experiment, the rats were kept fasting overnight and were deeply anesthetized with ketamine (60 mg/kg) and xylazine (7.5 mg/kg) [42, 43]. Blood samples were collected from each animal through aortic punctures for the preparation of serum. Liver tissues were carefully removed on ice-cooled glass plates and rapidly weighed and isolated prior to being stored in liquid nitrogen for further biochemical analyses. In order to determine the Al content in the tissue and perform the histological analyses, an adequate part of the liver tissue was promptly fixed in formalin 10% for 48 h. A portion of the liver tissue was also used to determine the mRNA (HO-1 and MT-1) and protein (CYP450) expression.

#### Hepatic marker enzyme assay

Serum alanine aminotransferase (ALT) and plasma aspartate aminotransferase (AST) activities were measured calorimetrically using the diagnostic kits (Pars Azmoon company, Tehran, Iran); according to the method determined by Reitman and Frankel [44].

#### Oxidant and antioxidant enzymes assay

The liver was homogenized in phosphate-buffered saline (PBS) solution (≈300 mg tissue per 3 mL PBS; pH 7.4). The homogenates were centrifuged at 4000 rpm for 10 min at 4 °C based on the kit protocol. The supernatant was divided into aliquots and then used to determine, MDA, iNOS, CAT, T-SOD, GPx, and TAC activities according to the protocols of the kits [45, 46].

#### Liver mRNA expression assay

To obtain the gene expression profile, total RNA was extracted from the liver tissue samples using Trizol reagent in accordance with the manufacturer's protocols. Next, cDNA was synthesized from total RNA using a High-Capacity cDNA Reverse Transcription Kit (Thermo Scientific, USA). Finally, real-time reverse transcription-PCR (qRT-PCR) was performed utilizing an automated sequence detection method (ABI Stepone, USA). HO-1, MT-1 and GAPDH mRNA expression levels were determined using SYBR Green qPCR Master Mix (addbio, Korea). The relative mRNA expression levels were determined using the Pfaffl method [47]. The sequences of primers are provided in Table 1.

**Table 1:** RT-PCR primers the Gen Bank accession numbers

Gene	Forward (F)/Reverse (R) Primers	Accession No. Gene Bank	Location	RT-PCR product (bp)
**HO-1**	F: TGACCATGACTGCTTTCCCCC R: ACCCCTCAAAAGACAGCCCTAC	NM_012580.2	1300–1320 1476–1455	177
**MT-1**	F: AGGGCTGTGTCTGGAAAGGTG R: AGGAAACTGGGTGGAGGTGT	NM_138826.4	206–226 340–321	135
**GAPDH**	F: AGGTCGGTGTGAACGGATTTG R: TGTAGACCATGTAGTTGAGGTCA	NM_017008.4	83–103 205–183	123

#### Liver protein expression assay

The immune-histochemical method was used for determining the expression levels of CYP450 protein enzymes in the liver tissue. The tissue was fixed in 10% formalin and underwent dehydration, blocking, and sectioning stages. Silane-coated slides holding the samples were then placed in a microwave oven and heated until boiling point was reached, after which the oven was turned off. The samples were washed with PBS in three steps, followed by permeabilization of the membrane using 3% Triton and blocking of the secondary antibody reaction with 10% goat serum. Primary and secondary antibodies were added according to the protocol [48], with the secondary antibody labeled with Alexa Fluor 488. Subsequently, DAPI was applied to the samples, and images of the desired markers were captured using a fluorescence microscope. The cells that exhibited a response to the green marker were quantified relative to the total number of blue-stained nuclei using Image J software. The data were analyzed using Prism software, and the percentage of CYP450 protein expression in the liver tissue was determined.

### Al content in the liver tissue

Liver samples weighing 0.2 g were digested using a solution of 65% HNO_3_ (Merck, Germany) and 2 mL of deionized water The mixture was vigorously mixed and Al concentrations were determined using inductively coupled plasma mass spectrometry (ICP-MS) as previously described [42, 43]. The settings and the operating conditions for ICP-MS during measurements are shown in Table 2.

**Table 2:** ICP-MS operating conditions

Parameters	Value / type
RF generator Power	1200 W
RF frequency	Resonance frequency: 24 MHz
Plasma, auxiliary, and nebulizer gas	Argon
Plasma gas flow rate	12.2 (L/min)
Auxiliary gas flow rate	0.8 (L/min)
Nebulizer gas flow rate	0.8 (L/min)
Sample uptake time	260 total (S)
Measurement replicate	3
Type of detector Solid state	CCD
Type of spray chamber cyclonic	Modified Lichte

### Histological assay

Liver tissue fragments were preserved in 10% formalin for 48 h. The fixed tissues were then embedded in paraffin blocks before being sectioned into 5μm slices using a microtome. Subsequently, the sections were stained with hematoxylin and eosin (H&E), following previously described methods [49]. The stained sections were examined using light microscopy with an × 40 magnification lens (LABOMED). To assess liver tissue injury in a semi-quantitative manner, the Mann et al. method was employed. In this method, a score of 0 indicates no change, while scores ranging from 1 to 4 indicate increasing severity of injury [50].

### Statistical analysis

GraphPad Prism 9 was employed to statistically analyse significant differences between treated and control rats (GraphPad Software, San Diego, CA, USA). The normality of the data was assessed using the Shapiro–Wilk test. The data were also analysed using One way and Two-way ANOVA, followed by the Tukey's Multiple Comparison test. The findings were presented as mean ± standard deviation (M ± SD) and a probability value of *P* < 0.05 was considered statistically significant.

## Results

### Morphological Characterization of Al_2_O_3_ NPs

**Fig. 1:**
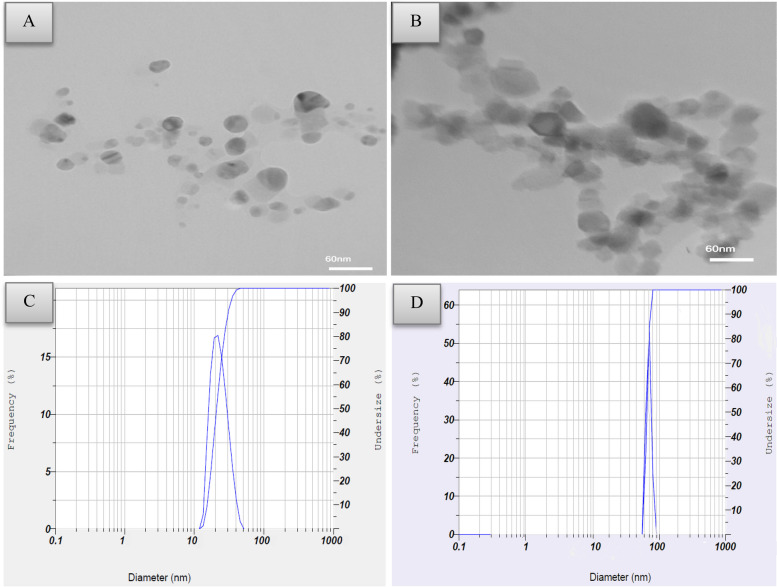
(**A**) TEM image of γ-Al_2_O_3_ NPs, (**B**) TEM image of α-Al_2_O_3_ NPs, (**C**) Particle size distribution of γ-Al_2_O_3_ NPs by DLS, (**D**) Particle size distribution of α-Al_2_O_3_ NPs by DLS

**Fig. 2:**
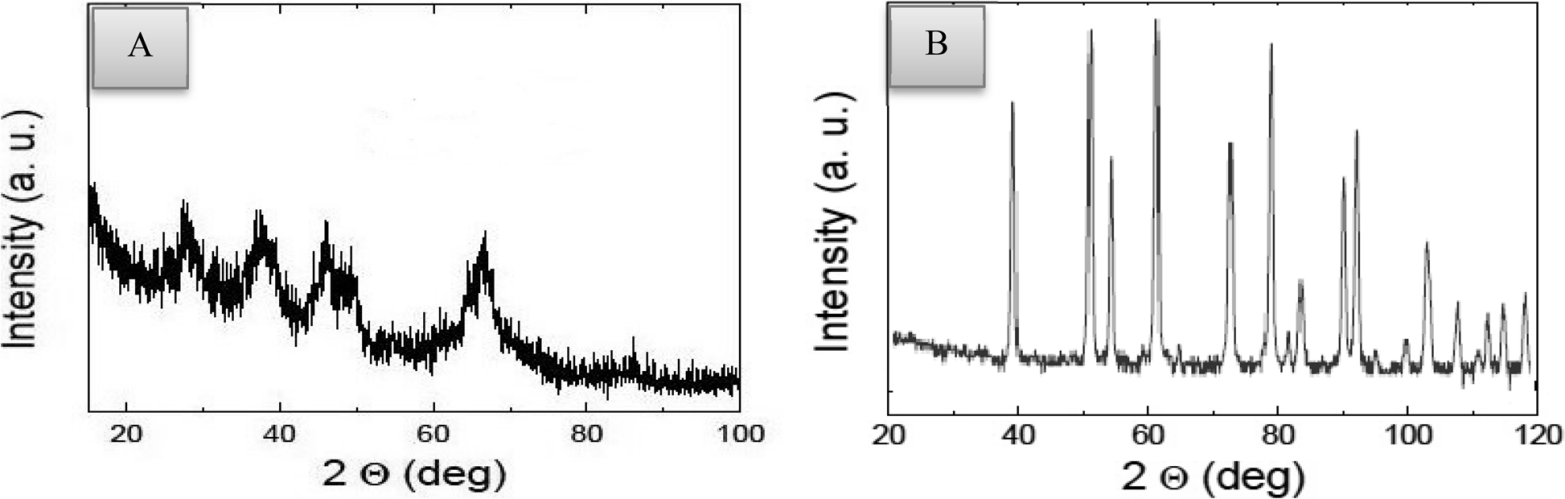
X-Ray Diffraction (XRD) of Al_2_O_3_-NPs; (**A**) γ-Al_2_O_3_ NPs with a size of 20 nm, (**B**) α-Al_2_O_3_ NPs with a size of 80 nm

Transmission electron microscopy (TEM) images (Fig. 1A and B) revealed the morphology of Al_2_O_3_ NPs (α-Al_2_O_3_ 80 nm and γ-Al_2_O_3_ 20 nm). The findings showed that the alpha phase had a rhombohedral structure morphology with an average diameter of 80 nm (Fig. 1B) while the gamma phase exhibited a roughly spherical morphology with an average diameter of 20 nm (Fig. 1A). According to the results from DLS, the particle size of γ-Al_2_O_3_ NPs was found to be 20 ± 6.3 nm, while α-Al_2_O_3_ NPs had an average diameter of 80 ± 4.3 nm (Fig. 1C and D). The XRD analysis (Figs. 2A and B) showed dominant peaks, confirming the crystalline nature of the Al_2_O_3_ NPs.

### DPPH radical-scavenging activity

The DPPH assay showed that the extract's radical-scavenging activity increased with higher concentrations. IC50 for DPPH radical-scavenging activity was 115 ± 30.6 µg/ml. The IC50 values for Quercetin and BHA were 1.336 ± 0.11, and 13.49 ± 1.04 µg/ml, respectively.

### Assessment of body and liver weight

Mortality was not observed among the rats during the research period. No significant changes were observed except for the group supplemented with a concentration of Abs, while rats administered with γ-Al_2_O_3_ showed a significant decrease in body weight gain (*P* = 0.001), relative body weight gain (*P* = 0.003), liver weight (*P* = 0.001) and relative weight of liver to body weight (*P* = 0.01) when compared with the control group (Table 3). Rats treated with α-Al_2_O_3_ showed a significant decrease in both body weight gain (*P* = 0.048) and liver weight (*P* = 0.016) compared to the control rats. When compared to the controls, a significant decrease (*P* = 0.035) was observed in the values of body weight gain among the rats treated with γ-Al_2_O_3_ plus Abs. The liver weight of the rats in the α-Al_2_O_3_ plus Abs group was less than (*P* = 0.017) the control group. Nonetheless, no significant differences between rats from the control group and other treated groups were observed. Table 3 presents the values of body weight, liver weight, and the relative weight of the liver to body weight in the different groups.

**Table 3:** Body weight, liver weight and relative weight changes of the rats from control, Al_2_O_3_ (γ and α) and Abs supplemented groups

Group	Initial weight(g)	Final weight (g)	Body gain(g)	Gain relative weight	Liver weight(g)	Liver relative weight
Control	213.5 ± 17.77	246.5 ± 21.22	33 ± 6.29	13.34 ± 1,93	12.45 ± 1.16	5.05 ± 0.29
Abs	202 ± 9.59	224.5 ± 9	22.5 ± 9.93	9.95 ± 4.23	11.02 ± 1.18	4.89 ± 0.35
γ -Al_2_O_3_	192 ± 15.93	202.33 ± 22.28	10.33 ± 4.36^a^	4.77 ± 4.95^b^	8.47 ± 1.53^a^	4.17 ± 0.47^b^
γ-Al_2_O_3_ + Abs	198.67 ± 16.7	216 ± 17.45	17.33 ± 4.13^c^	8.02 ± 1.78	9.98 ± 1.52	4.61 ± 0.45
α-Al_2_O_3_	196.83 ± 17.8	214.83 ± 23.2	18 ± 8.22^c^	8.22 ± 2.89	9.52 ± 1.7^c^	4.41 ± 0.51
α-Al_2_O_3_ + Abs	186 ± 17.16	207.4 ± 17.7	21.4 ± 9.71	10.09 ± 4.31	9.83 ± 1.14^c^	4.71 ± 0.30

The presented data represent the mean ± standard deviation. Mean initial, final body weight, body gain, gain relative weight, liver weight and liver relative weight: *P* value was considered significant at ^a^*P* ≤ 0.001, ^b^*P* ≤ 0.01, and ^c^*P* ≤ 0.05 compare to control (One-way ANOVA)

### Assessment of liver function markers

**Fig. 3:**
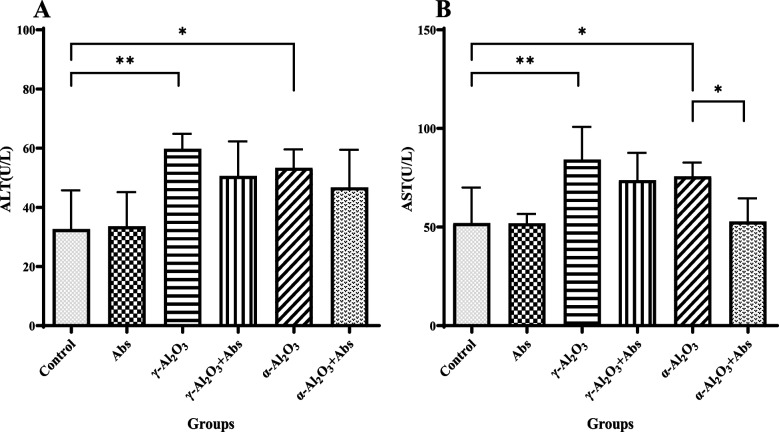
Illustrates the alterations in levels of (**A**) ALT activity and (**B**) AST activity. The values are presented as mean ± S.D (*n* = 6 per group). Statistical analysis using two-way ANOVA revealed ** *P* < 0.01 and * *P* < 0.05

Figure 3(A, B) shows the mean levels of ALP and ALT enzymes in groups that received Al_2_O_3_ NPs (γ and α) and were pre-treated with Abs. The mean activity of AST (84.27 ± 16.54 vs. 52.06 ± 17.96, *P* = 0.0019) and ALT (59.76 ± 5.05 vs. 32.69 ± 13.03, *P* = 0.0014) was significantly increased in serum after administration of γ-Al_2_O_3_ NPs compared to the control group. The α-Al_2_O_3_ NPs also contributed to significant increase in AST serum levels (75.73 ± 6.97 vs. 52.06 ± 17.96, *P* = 0.036) and ALT (53.38 ± 6.17 vs. 32.69 ± 13.03, *P* = 0.020) serum levels comparison to the control group. Moreover, pre-treatment with Abs significantly decreased the elevated AST activity caused by α-Al_2_O_3_ NPs (52.85 ± 11.67, *P* = 0.046). No significant differences were observed in AST, and ALT levels between the groups that had been pre-treated with Abs and received γ-Al_2_O_3_ NPs.

### Assessment of MDA and iNOS levels

**Fig. 4:**
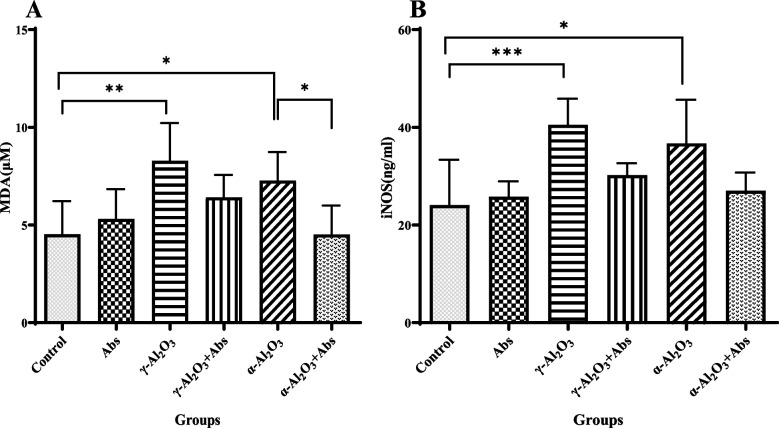
**A**, **B**. The levels of MDA (**A**) and iNOS (**B**) in the liver homogenate were compared among different groups. The data are presented as mean ± SD with a sample size of 6 (*n* = 6). Statistical analysis was conducted using a two-way ANOVA followed by Tukey's multiple comparison test. The significance levels were denoted as ***P < 0.001, **P < 0.01, and *P < 0.05

The levels of MDA and iNOS among different groups of rats are shown in Fig. 4 (A-B). The MDA level in liver tissues was significantly higher in the γ-Al_2_O_3_ NPs group (8.28 ± 1.93, *P* = 0.003) and α-Al_2_O_3_ NPs group (7.28 ± 1.45, *P* = 0.047) compared to the control group (4.53 ± 1.7). However, of Abs significantly improved the MDA level in the Abs plus α-Al_2_O_3_ NPs group (*P* = 0.046), particularly when compared to the α-Al_2_O_3_ NPs group. There were no significant differences in MDA levels among the other groups (Fig. 4A).

The levels of iNOS in liver tissues were significantly elevated in the γ-Al_2_O_3_ NPs group (40.53 ± 5.33, *P* < 0.001) and α-Al_2_O_3_ NPs group (36.71 ± 8.93, *P* = 0.013) compared to the control group (24.08 ± 9.23). However, treatment with Abs almost marginally decreased the level of iNOS among the rats from Abs plus Al_2_O_3_ NPs compared with Al_2_O_3_ NPs rats but not significantly (Fig. 4B).

### Assessment of T-SOD, CAT, GPx and TAC activity

**Fig. 5:**
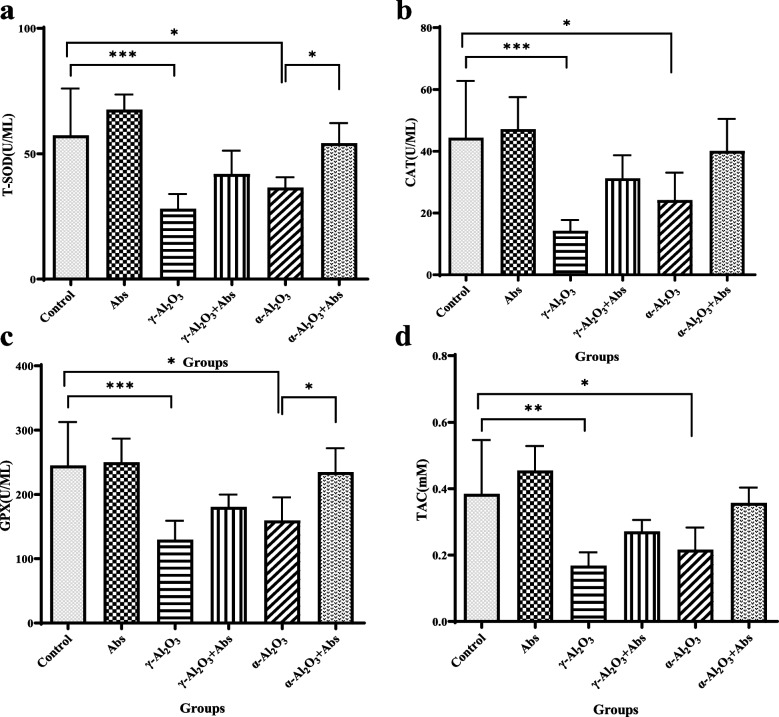
**a**-**d**. The levels of T-SOD (**a**), CAT (**b**), GPx (**c**), and TAC (**d**) in liver homogenates were analyzed. The data are presented as mean ± SD with a sample size of 6 (*n* = 6). Statistical analysis was conducted using a two-way ANOVA, followed by Tukey's post hoc test for multiple comparisons. Significant differences were denoted as *** *P* < 0.001, ** *P* < 0.01, and * *P* < 0.05

The levels of T-SOD, CAT, GPx, and TAC are shown in Fig. 5(a-d). The activity of T-SOD in the liver tissues of rats from the γ-Al_2_O_3_ NPs (28.06 ± 5.89, *P* < 0.001) and α-Al_2_O_3_ NPs (36.49 ± 4.16, *P* = 0.032) groups was significantly decreased compared to the control group (57.33 ± 18.71) (Fig. 5a). CAT activity in the liver tissues was also found to be significantly decreased in the γ-Al_2_O_3_ NPs (14.23 ± 3.52, *P* < 0.001) and α-Al_2_O_3_ NPs (24.18 ± 8.86, *P* = 0.012) groups compared to the control group (44.37 ± 18.39) (Fig. 5b). Similarly, the activity of GPx in liver homogenates was significantly decreased in the γ-Al_2_O_3_ NPs group (129.5 ± 29.47, *P* < 0.001) and the α-Al_2_O_3_ NPs group (159.4 ± 35.89, *P* = 0.011) compared to the control group (244.9 ± 67.61) (Fig. 5c). Additionally, the TAC activity was significantly reduced in both the γ-Al_2_O_3_ NPs group (0.168 ± 0.039, *P* = 0.001) and the α-Al_2_O_3_ NPs group (0.216 ± 0.066, *P* = 0.016) compared to the control group (0.38 ± 0.16) (Fig. 5d). In contrast, pre-treatment with Abs significantly suppressed the α-Al_2_O_3_ NPs-induced reduction in the activity of T-SOD (54.21 ± 8.04, *P* = 0.043) and GPX (234.5 ± 37.71, *P* = 0.034) (Fig. 5a-c). No significant differences were observed in the levels of antioxidant biomarkers between the Al_2_O_3_ NPs groups and the Abs plus Al_2_O_3_ NPs groups.

### Signalling pathway analysis

**Fig. 6:**
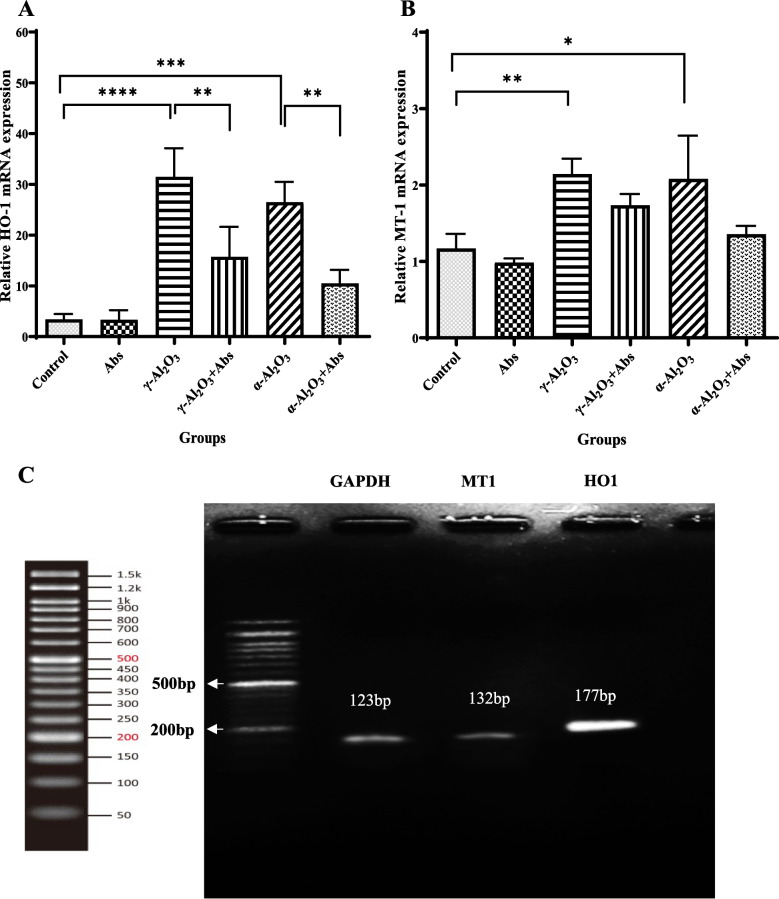
Effect of Abs on Al_2_O_3_ NPs-induced expression of HO-1 (**A**) and MT-1 (**B**) mRNAs. Size analysis of PCR products by agarose gel electrophoresis (**C**). The data are presented as mean ± SD. Statistical analysis was performed using two-way ANOVA followed by Tukey's post hoc test for multiple comparisons. The significance levels were denoted as ****P < 0.0001, ***P < 0.001, **P < 0.01, and *P < 0.05

HO-1 and MT-1 were identified as the main pathways activated by Al_2_O_3_ NPs and Abs during the treatment process (Fig. 6A–B). The analysis of PCR product sizes by agarose gel electrophoresis is depicted in Fig. 6c. Our findings demonstrated that injecting rats with γ-Al_2_O_3_ NPs significantly increased the expression of oxidative-related genes, specifically HO-1 (31.47 ± 5.63 vs 3.40 ± 1.08, *P* < 0.001) and MT-1 (2.14 ± 0.198 vs 1.170 ± 0.19, *P* = 0.008), compared to the control group. Also, Abs had no significant effects on MT gene expression in the groups exposed to Al_2_O_3_ NPs (α and γ). Furthermore, in the liver tissue of rats treated with α-Al_2_O_3_ NPs, the mRNA expression of HO-1 and MT-1 was significantly up-regulated compared to the control group, with values of 26.49 ± 3.99 vs 3.40 ± 1.08, *P* = 0.001 and 2.08 ± 0.56 vs 1.170 ± 0.19, *P* = 0.013, respectively. However, pre-treatment with Abs notably down-regulated the mRNA expression of HO-1 in rats treated with Abs plus γ-Al_2_O_3_ NPs (15.74 ± 5.9, *P* = 0.0036) and Abs plus α-Al_2_O_3_ NPs (10.52 ± 2.66, *P* = 0.004) compared to rats solely treated with Al_2_O_3_ NPs (α and γ).

### Assessment of protein expression of CYP450 enzyme

**Fig. 7:**
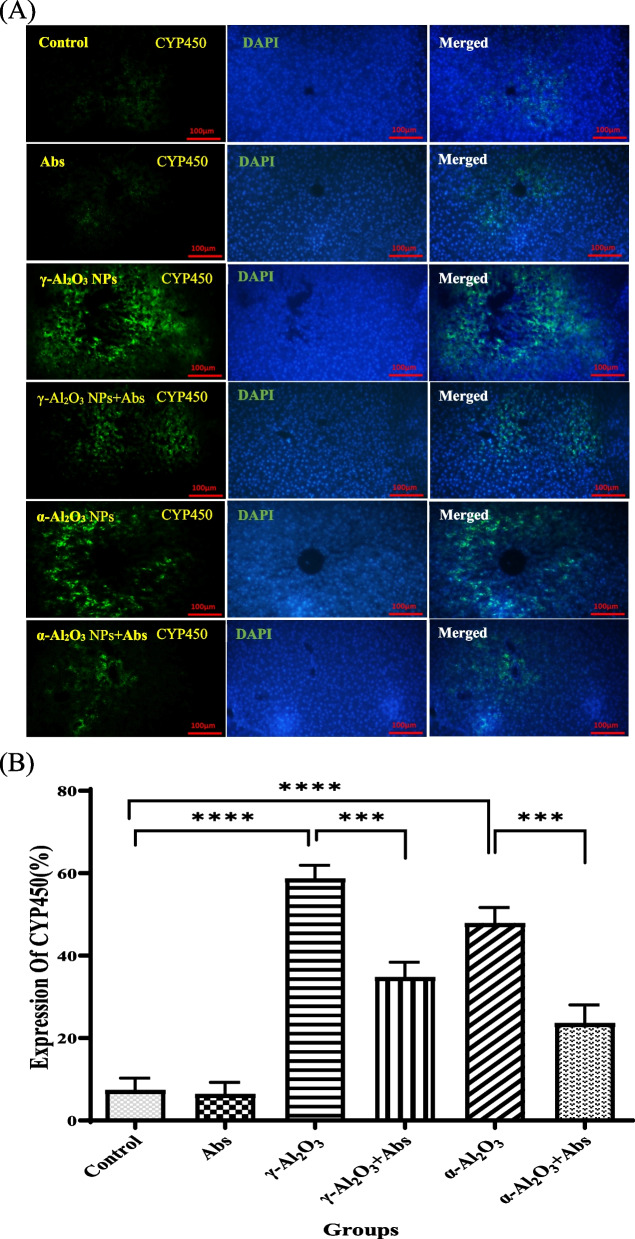
The protein levels of CYP450 were analyzed using immune histochemical techniques. (**A**) CYP450 were stained with green color, cell nucleus was labelled by DAPI with blue color and then merged. Images indicated the positions of CYP450/DAPI/merged. Scale bars = 100 μm. (**B**) Analysis of CYP450 expression. ***P < 0.001, ****P < 0.0001. Data are shown as mean ± SD (*n* = 6 per group). Data were analysed by two-way ANOVA test

The results of the immune-histochemical analyses showed a significant up-regulation (*P* < 0.001) of CYP450 protein expression in the rat liver after treatment with α-Al_2_O_3_ NPs (47.81 ± 2.22) and γ-Al_2_O_3_ NPs (58.63 ± 1.90), compared to the control group (Fig. 7A). The rats treated with Al_2_O_3_ NPs exhibited strong immunostaining of CYP450, which was diffusely distributed in the hepatocytes (Fig. 7B). Moreover, pre-treatment with Abs significantly down-regulated the protein expression of CYP450 in the Abs plus γ-Al_2_O_3_ NPs (34.76 ± 2.09) and Abs plus α-Al_2_O_3_ NPs (23.60 ± 2.58) groups, compared to rats treated only with Al_2_O_3_ NPs (α and γ) (*P* < 0.01) (Fig. 7A). The staining intensity was reduced in the rats pre-treated with Abs, as compared to the other groups (Fig. 7B). (A).

### Assessment of Al content

**Fig. 8:**
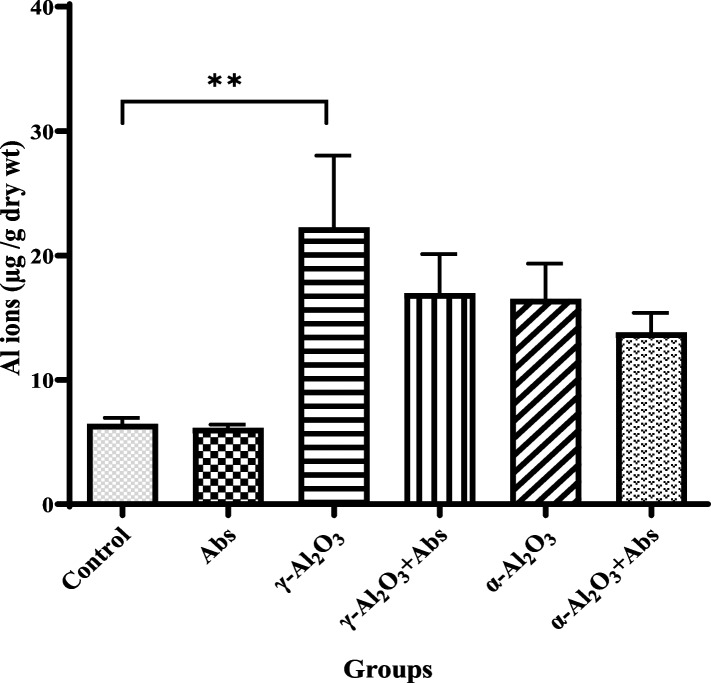
The effects of Al_2_O_3_ NPs (α and γ) on tissue Al concentration were investigated, as well as the potential pre-treatment effects of Abs on this concentration. The values are expressed as mean ± SD (*n* = 6). Statistical analysis was conducted using one-way ANOVA, and the significance level was indicated as **P < 0.01

Figure 8 illustrates that the average aluminum concentration in the liver of rats treated with γ-Al_2_O_3_ nanoparticles (20 nm size) was significantly higher (22.27 ± 5.78, *P* = 0.009) compared to the control group (6.46 ± 0.48). In the groups that received α-Al_2_O_3_ nanoparticles (80 nm size), there was an increase in the concentration of these nanoparticles in the liver tissue, but it did not reach statistical significance. Pre-treatment with Abs mitigated the increase in Al concentration in the groups that were administered Al_2_O_3_ NPs, although the effect was not statistically significant.

### Histopathological evaluation

**Fig. 9:**
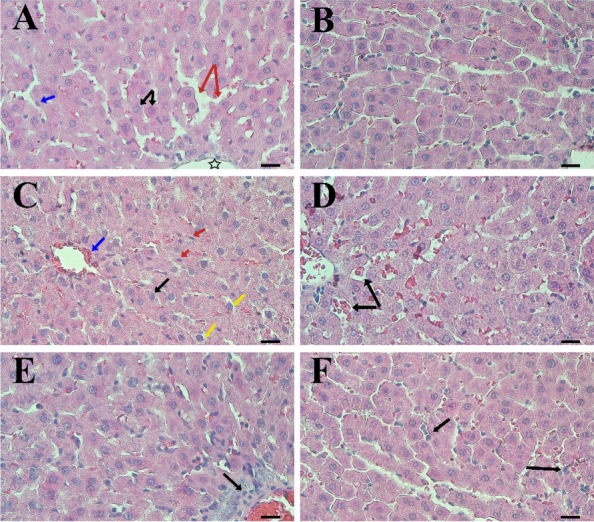
Histopathological examination of liver sections from rats in different groups was conducted. Group **A** (control) and Group **B** (Abs) showed light micrographs of rat liver sections. The histology of the control group exhibited normal architectures, including a central vein (star), sinusoids (red arrows), Kupffer cells (blue arrow), and hepatocytes arranged in hepatic cords (black arrows). In Group **C** (γ-Al_2_O_3_), the liver section displayed a central vein (CV) containing hemolysed red blood cells (blue arrow). The structure of hepatic cords and sinusoids was disrupted. Hepatocytes had dark nuclei (black arrow), and there was an increase in Kupffer cells (red arrows). Group **D** (Abs plus γ-Al_2_O_3_) showed a significant decrease in tissue disruption. Some areas exhibited dilated sinusoids (arrows). Accumulation of blood cells in the central vein and sinusoids was observed. In Group **E** (α-Al_2_O_3_), the liver section revealed a clearly congested thick-walled central vein containing hemolysed blood cells and extensive inflammatory cell infiltration (black arrow). The structure of hepatic cords was disrupted. Group **F** (Abs plus α-Al_2_O_3_) displayed a slightly increased number of Kupffer cells and hepatocytes (arrows) in the rat's liver section.Scale bars: 20 µm (40X)

The examination of samples from rats in the control group using a light microscope revealed normal liver architecture. Each lobule had a central vein, and the hepatic cords were arranged in a radiating shape (Fig. 9A). In the Abs group, the morphology was virtually identical to the control group, demonstrating a normal structure (Fig. 9B). However, of γ-Al_2_O_3_ NPs resulted in evident hepatic alterations. These included distorted hepatic structure, dilatation, congestion of the central vein and hepatic sinusoids. The diameter of some hepatocytes was smaller than that of neighboring cells, and activated Kupfer cells were observed compared to the control group. Accumulation of blood cells was also observed in the central vein and sinusoids. Additionally, dark nuclei of varying sizes were present (Fig. 9C). On the other hand, these alterations were less pronounced in the Abs plus γ-Al_2_O_3_ NPs group. The examined sections from this group showed mild morphological changes, limited to slight dilatation of the sinusoids and accumulation of blood cells in the central vein. The radial arrangement of hepatic cords and sinusoids was superior to that of rats receiving γ-Al_2_O_3_ NPs (Fig. 9D). Notably, 14 days after exposure to α-Al_2_O_3_ NPs, liver damage was evident. Inflammatory cell infiltration was observed around the blood vessels, and degradation of hepatocytes and nucleoli were unclear. Similarly, blood cell and inflammatory cell infiltration were observed around the central vein. The hepatic cord structures were disrupted, although the boundaries of the hepatocyte cells could still be distinguished (Fig. 9E). In the Abs plus α-Al_2_O_3_ NPs group, the number of hepatocytes returned to normal levels, and there was a significant improvement in radial arrangement compared to the rats receiving α-Al_2_O_3_ NPs. The histological alterations caused by α-Al_2_O_3_ NPs were also significantly reduced in the liver of this group. Abs preserved an almost normal structural pattern with better cord arrangement of hepatocytes. There was a slight increase in Kupffer cells compared to the group that received α-Al_2_O_3_ NPs. Some hepatocytes exhibited normal-sized nuclei, while others displayed shrunken nuclei (Fig. 9F).

**Fig. 10:**
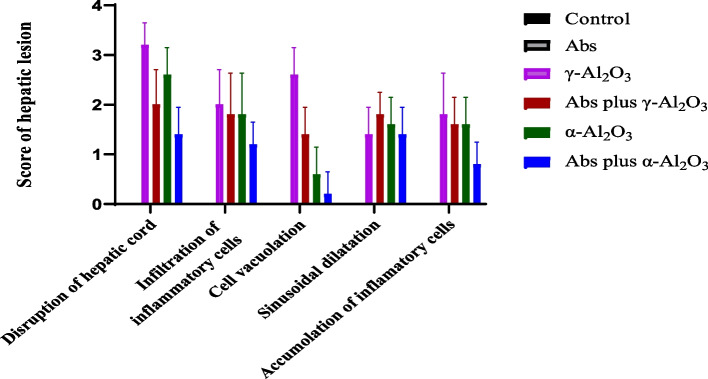
Scoring of hepatic damage

The scoring of liver damage revealed that in the γ-Al_2_O_3_ NPs group, liver tissue damage progressed to the severe form (score 4). In the α-Al_2_O_3_ NPs group, the severity of damage ranged from slight (score 2) to moderate (score 3); however, in other groups, it did not exceed the slight level (Fig. 10).

## Discussion

Nanoparticles made of aluminium oxide, specifically Al_2_O_3_ NPs, are widely used in the production of medicinal and commercial products. However, the concerns have been raised regarding the potential risks they pose to human health. This study aimed to investigate the adverse effects of Al_2_O_3_ NPs on various markers in the liver of rats, focusing on the hypothesis of shape-dependent hepatotoxicity between γ-Al_2_O_3_ NPs (20 nm) and α-Al_2_O_3_ NPs (80 nm).

The findings of this study revealed that γ-Al_2_O_3_ NPs (20 nm) were more hepatotoxic compared to α-Al_2_O_3_ NPs (80 nm), which can be attributed to the size difference between these nanoparticles. The hepatotoxicity resulted in weight changes and biochemical, gene expression and functional/structural alterations in hepatic tissues of the rats. Interestingly, it was hypothesized that Abs, a plant antioxidant, could ameliorate the hepatotoxicity caused by Al_2_O_3_ NPs, particularly α-Al_2_O_3_ NPs.

The showed that except for the α-Al_2_O_3_ NPs plus Abs and Abs groups, all other groups exhibited decreased body weight gain compared to the control group. This decrease in weight gain may be attributed to increased intracellular reactive oxygen species (ROS) production and oxidative stress, leading to anorexia or reduced food absorption. Previous studies have also reported weight loss or decreased weight gain as indicators of toxicity [51]. Another similar study reported that treatment of the rats with Al_2_O_3_ NPs (50nm, orally, 70 mg/kg/bw) for 75 days, contributed to significant decreased body weight gain [52]. Additionally, several studies have demonstrated the protective role of Abs against liver injury induced by chemicals or toxins through scavenging ROS and improving antioxidant capacity [23, 37, 53]. In this study, pre-treatment with Abs showed a protective effect against acute liver injury induced by Al_2_O_3_ NPs, potentially due to increased antioxidant capacity, which improves appetite, digestion, food intake, and metabolism. Consequently, Abs-treated rats exhibited an increase in relative liver weight and weight gain, indicating improved liver function and a return to normal conditions.

In the groups exposed to γ-Al_2_O_3_ NPs (20 nm), body weight gain and relative body weight gain were significantly decreased compared to the control group, suggesting that this specific form and size of Al_2_O_3_ NPs induce more damage in the liver than α-Al_2_O_3_ NPs.

The results also indicated that Al_2_O_3_ NPs caused elevated levels of MDA and iNOS primarily due to oxidative stress. This led to increased leakage of ALT and AST into bloodstream, indicating liver damage. Furthermore, the activity of antioxidant enzymes such as GPx, SOD, CAT, and TAC was decreased in rats treated with Al_2_O_3_ NPs, suggesting an impaired antioxidant system and amplified oxidative stress. Notably γ-Al_2_O_3_ NPs with a spherical shape and size of 20 nm, had a significant impact on these markers.

Consistent with these findings, previous studies have reported that Al_2_O_3_ NPs induced an increase in AST, ALT, and MDA levels as well as a decrease in TAC, CAT, SOD, and GPx activities [12, 54, 55]. However, no previous research has explored the contribution of Abs to the changes in liver functionality and biochemical markers induced by Al_2_O_3_ NPs. Pre-treatment with Abs increased the activities of T-SOD, GPX, CAT, and TAC while decreasing MDA and iNOS in rats exposed to Al_2_O_3_ NPs. This suggests that Abs can mitigate the toxic effects of Al_2_O_3_ NPs on these enzymes by acting as an antioxidant and scavenging reactive oxygen and nitrogen free radicals [56].

Phytochemically, Abs has been reported to possess essential oil, absinthin, anabsin, anabsinthin, artabsin and matricin; resins, lactones and organic acids [26]. Abs also contains flavonoids such as quercetin, rutin and other flavonoid glycosides (isoquercitrin, quercitin-3-O–d-glucoside, quercitin-3-O-rhamnoglucoside, isorhamnetin-3-O-rhamnoglucoside, isorhamnetin-3-glucoside), as well as phenolic acids such as chlorogenic, syringic, coumaric, salicylic and vanillic acids and organic acids. Furthermore, Abs may have additional components, all of which have the potential to inhibit free radicals [26].

The Abs hepatic protection can be related to chlorogenic acid and Quercetin derivatives identified in its aqueous extract [57]. Additionally, sesquiterpenes extracted from Abs and flavonoids from Artemisia species have demonstrated anti-inflammatory effects. Furthermore, tetra monoxido hydroxyflavone extracted from Absinthium has been found to inhibit inflammatory mediators. These pharmacophores contribute to the antioxidant and anti-inflammatory activities of Abs [26].

HO‑1 and MT‑1 genes are rapidly upregulated by pro- inflammatory cytokines or oxidative stress, as a protection mechanism against cellular stress in the liver [58]. HO-1 induction is typically a response to counteract cellular stress. However, the products of HO-1 can be protective at lower doses but cytotoxic at higher doses. The optimal duration of HO-1 induction is challenging to determine as sustained expression can be harmful, while short-lived expression may be ineffective [59]. Horie et al. reported an increase in relative levels of the HO-1 gene following the intratracheal instillation of nano-NiO [60]. MT-1 helps mitigate the effects of oxidative stress by scavenging free radicals or preventing their formation. In our study, we observed that Al_2_O_3_ NPs significantly increased the expression of HO-1 and MT-1 mRNAs in the liver tissues of rats. However, pre-treatment with Abs effectively down-regulated this expression in a size-dependent manner. Alghriany et al. [54] demonstrated that oral administration of Al_2_O_3_ and Al_2_O_3_ NPs at a dose of 6 mg/kg/bw increased the p53 and Nrf2 levels while decreasing the level of Hsp70. In line with our observations, co-administration of nano curcumin with Al_2_O_3_ NPs improved these changes. Similarly, the genes HO-1 and MT-1 are significantly enhanced in response to oxidative stress and pro-inflammatory cytokines as a protective mechanism against cellular stress in the liver [58].

In our results, expression levels of HO-1 and MT-1 were regulated significantly by Al_2_O_3_ NPs. These changes are closely related to the up-regulation of CYP 450 enzymes in the liver. CYP450 enzymes are monooxygenases encoded by P450 genes and are primarily found as membrane-bound proteins in the endoplasmic reticulum of the liver. These enzymes evolved as the primary defense against a wide range of endogenous and exogenous compounds, including the bioactivation of toxicants to more reactive intermediates [61]. Additionally, CYP 450 enzymes convert hydrophobic compounds into hydrophilic compounds to facilitate their excretion [62]. Our study indicated an up-regulation of CYP450 in hepatic cells exposed to γ-Al_2_O_3_ NPs and α-Al_2_O_3_ NPs. Notably, the expression of CYP 450 protein was higher in the group exposed to γ-Al_2_O_3_ NPs, as confirmed by immune-histochemical images showing a greater increase in staining intensity. Oxidative stress plays a crucial role in influencing CYP450 expression in the liver [63, 64]. Pre-treatment with Abs, known for its antioxidant effects, reduced the expression of CYP450 protein in the groups receiving Al_2_O_3_ NPs, as supported by immune-histochemical images. Signaling pathways involving oxidative, inflammatory, and nuclear receptors also interact to influence the expression of CYP450 enzymes [65]. Previous research has shown that oxidative stress, particularly through the activation of nuclear receptor signaling pathways, can regulate CYP450 expression [66].

In our opinion, Al_2_O_3_ NPs injected in the systemic pathways are absorbed by the liver. Our findings suggest that Al_2_O_3_ NPs enhance the bioaccumulation of aluminum in the liver. The results demonstrated elevated levels of aluminum in the liver tissue of rats exposed to γ-Al_2_O_3_ NPs, potentially leading to increased toxicity at the cellular level. Due to the small particle size (20 nm) and specific phase (γ) of Al_2_O_3_ NPs, these particles can easily access various parts of the body, including the liver, through blood circulation and accumulate there.

Pre-treatment with Abs contributed to a decrease in liver Al content, which may be attributed to Abs’ ability to scavenge ROS. Several in vivo studies have reported that Al accumulation in the brain [67, 68], and hippocampus [69] can result in neurodegenerative and neurological disorders [70]. Moreover, there is a strong association between metal concentration in tissues and liver damage, as demonstrated by previous studies [71].

Histopathological examination revealed that the injection of Al_2_O_3_ NPs induced inflammation, structural destruction, an increase in Kupffer's cell, extensive infiltration of inflammatory cell, and disorder of hepatic cords and sinusoids. Moreover, liver tissue was more severe in rats exposed to γ-Al_2_O_3_ NPs comparison to α-Al_2_O_3_ NPs. Previous studies have reported that oral administration of Al_2_O_3_ NPs can lead to liver inflammation, hepatocyte necrosis, blood sinusoids damage, and fibrosis [54]. Hadi and Jaffat et al. also observed morphological changes in the liver, testes, and kidneys following exposure to Al_2_O_3_ [72]. Similarly, Canli et al. mentioned that Al-NPs caused hepatic necrosis, and disarray, and cell − cell dissociation [73]. Our results demonstrated that oral administration of Abs mitigated liver damage. These findings are consistent with Alghriany et al. findings that Curcumin may alleviate pathological injury caused by Al_2_O_3_ NPs in hepatic tissue [54].

The current study had several limitations, which are outlined below:

1. One of the main limitations of this study was the selection of a single dose of Abs, which had been determined as the most effective dosage in previous studies. Due to constraints, the most effective dosage was selected based on the reference of maximum protection against liver damage with a dose of 200 mg/kg body weight [23, 37, 38]. However, it is recommended to investigate alternative dosages in future studies as well.
2. Another limitation of this study is that it only utilized two specific forms and sizes of Al_2_O_3_ NPs through intraperitoneal administration. Further studies with different forms and other routes of administration (such as inhalation) could be performed in the future and may lead to different results.

## Conclusion

The finding of this research indicates that acute exposure to Al_2_O_3_ NPs induces oxidative stress and damage in liver tissues. The phase of Al_2_O_3_ NPs plays a crucial role in their uptake and interaction with biological tissues, resulting in adverse effects. Accordingly, in the present study, the toxicity of Al_2_O_3_ NPs was compared based on their phases. Our findings revealed that administration of the γ-Al_2_O_3_ NPs to the rats caused more potent and harmful alterations than α-Al_2_O_3_ NPs. In addition, pre-treatment with an aqueous extract of Abs would effectively protect and prevent the adverse effects and oxidative stress induced by Al_2_O_3_ NPs in the liver tissues of the rats. Nonetheless, the specific pathway by which Al_2_O_3_ NPs contribute to toxicity remains unknown, and additional studies are required to precisely characterize this mechanism. Consequently, it is recommended that a significant deal of focus should be placed on the possible beneficial health effects of plant antioxidants, not only to manage cellular oxidative damage but also to reduce the toxicity of NPs.

## Data Availability

The data used to support the findings of this study have been included in this article. Additional files are available from the corresponding authors upon request.

## Abbreviations

Al_2_O_3_ NPs: Oxide Aluminium nanoparticles
Abs: *Artemisia **Absinthium* L.
γ: Gamma
α: Alpha
MDA: Malondialdehyde
GPx: Glutathione peroxidase
iNOS: Inducible nitric oxide synthase
ALT: Alanine transaminase
AST: Aspartate aminotransferase
CAT: Catalase
T-SOD: Total superoxide dismutase
TAC: Total antioxidant capacity
HO-1: Heme oxygenase-1
MT-1: Metallothionein-1
CYP450: Cytochrome P450
ROS: Reactive oxygen species
RNS: Reactive nitrogen species
TEM: Transmission electron microscopy
XRD: X-ray diffraction
DLS: Dynamic light scattering
DPPH: Radical 1, 1-diphenyl-2-picryl hydrazyl
BHA: Butylated hydroxyanisole
ICP-MS: Inductively coupled plasma mass spectrometry
